# Heat stress in horses: a literature review

**DOI:** 10.1007/s00484-023-02467-7

**Published:** 2023-04-15

**Authors:** Hyungsuk Kang, Rebeka R. Zsoldos, Albert Sole-Guitart, Edward Narayan, A. Judith Cawdell-Smith, John B. Gaughan

**Affiliations:** 1 School of Agriculture and Food Sciences, The University of Queensland, Gatton, QLD 4343 Australia; 2 School of Veterinary Science, The University of Queensland, Gatton, QLD 4343 Australia

**Keywords:** Horse, Heat stress, Body temperature measurement, Cool-down strategy

## Abstract

Healthy adult horses can balance accumulation and dissipation of body heat to maintain their body temperature between 37.5 and 38.5 °C, when they are in their thermoneutral zone (5 to 25 °C). However, under some circumstances, such as following strenuous exercise under hot, or hot and humid conditions, the accumulation of body heat exceeds dissipation and horses can suffer from heat stress. Prolonged or severe heat stress can lead to anhidrosis, heat stroke, or brain damage in the horse. To ameliorate the negative effects of high heat load in the body, early detection of heat stress and immediate human intervention is required to reduce the horse’s elevated body temperature in a timely manner. Body temperature measurement and deviations from the normal range are used to detect heat stress. Rectal temperature is the most commonly used method to monitor body temperature in horses, but other body temperature monitoring technologies, percutaneous thermal sensing microchips or infrared thermometry, are currently being studied for routine monitoring of the body temperature of horses as a more practical alternative. When heat stress is detected, horses can be cooled down by cool water application, air movement over the horse (e.g., fans), or a combination of these. The early detection of heat stress and the use of the most effective cooling methods is important to improve the welfare of heat stressed horses.

## Introduction

There are more than 16.9 million horses in the world (Cross 2019) and approximately 0.24 million horses are registered globally for Thoroughbred racing (International Federation of Horseracing Authorities 2019), 11,444 horses globally for American Quarter Horse racing (American Quarter Horse Association 2021), and approximately 0.27 million horses are registered to compete at equestrian events (Fédération Équestre Internationale 2022). These include endurance, jumping, dressage, para-dressage, eventing, driving, vaulting, and reining. Given this substantial number of competition horses, it is not surprising that welfare issues have been raised. Heat stress is a serious welfare issue, not only during horse competition including Thoroughbred and Standardbred racing, endurance events, Olympic competition, and other equestrian disciplines, but also leisure riding, transportation, and inappropriate housing and management under hot and humid conditions (Waran et al. 2007; Pritchard et al. 2006; Brownlow et al. 2016; Padalino et al. 2016; Brown-Brandl et al. 2005; Holcomb et al. 2014). Even though there are no clear data of economic losses in the equine industry related to heat stress, extremely hot and humid climate conditions have detrimental effects on the industry by reducing athletic and reproductive performance, increasing the risk of infectious and heat stress–related diseases and injury, and affects equestrian event management (Melissa 2011).

Although heat stress is an important welfare issue, there is not a clear definition of heat stress in horses, and there is little data available regarding this condition. In relation to welfare in horses, heat stress can be defined as the inability of the horse to maintain body temperature within a prescribed temperature range (Caulfield et al. 2014; Marlin 2009; Spedding 2000). Understanding the impact of heat stress on horses will allow mitigation strategies to be developed and reduce the likelihood of adverse events across all levels of the equine industry. To help understand and improve the welfare of heat-stressed horses, we have used peer-reviewed publications papers to summarize information about what defines heat stress in horses and how to prevent injury and illness resulting from severe heat stress.

## Thermoregulation in horses

### Physical heat transfer

**Fig. 1:**
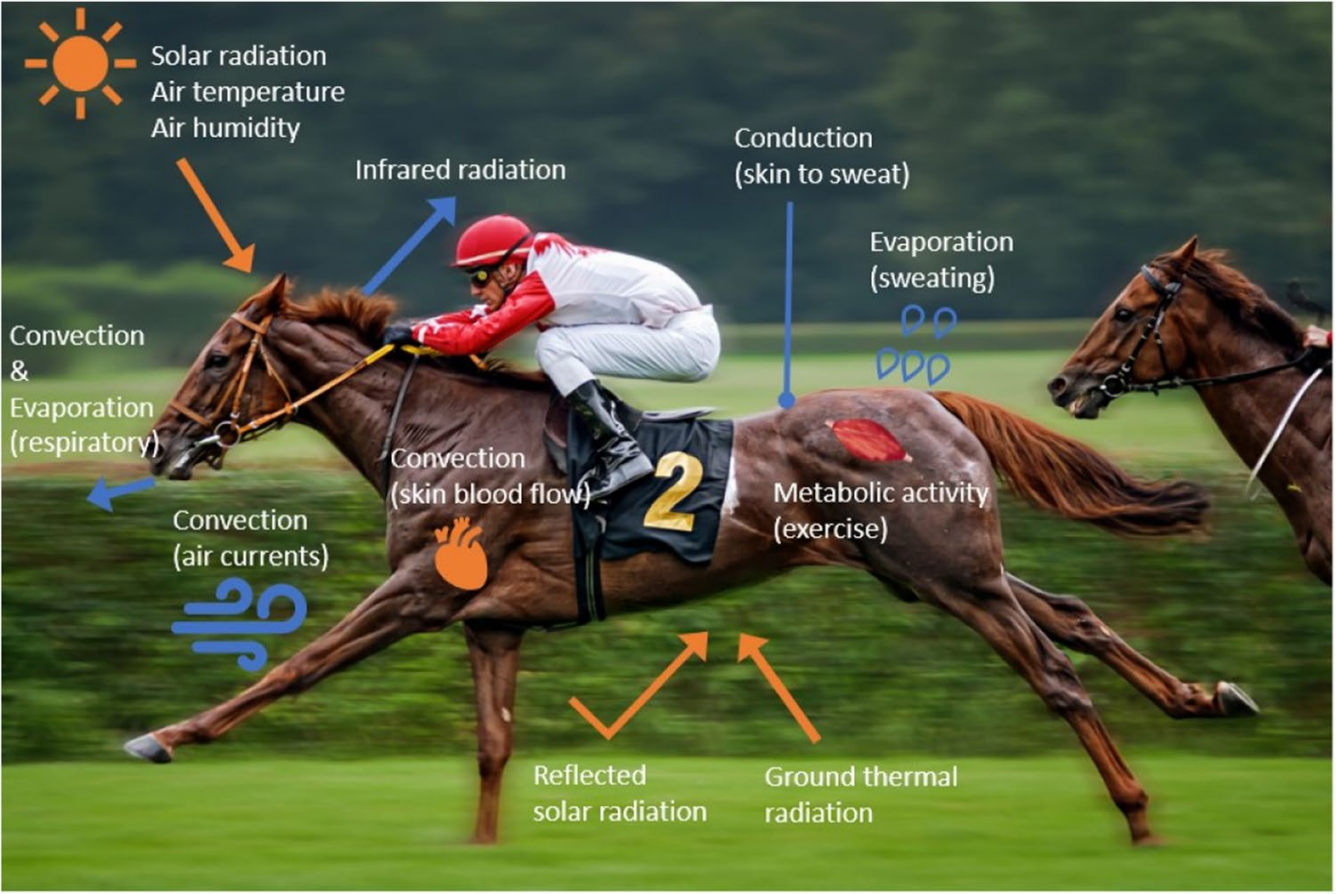
Physical heat transfer in exercising horses. Blue colour = heat dissipation; orange colour = heat accumulation. This image was adapted from open source ‘Adobe stock images’ and modified by Hyungsuk Kang

The normal body temperature range of a healthy horse is between 37.5 and 38.5 °C (Mealey 2019), when horses are in their thermoneutral zone (5 to 25 °C) (Morgan 1998). The body temperature of horses also fluctuates due to circadian and seasonal rhythms, where the minimum body temperature occurs in the early morning during the winter season and the maximum in the late afternoon during summer (Ayo et al. 2014; Giannetto et al. 2012; Kaseda and Ogawa 1993). Even though the body temperature fluctuates, keeping a balance between heat production (gain) and heat dissipation (loss) is essential to maintain the body temperature in a narrow range, and to avoid both cold stress (Mejdell et al. 2020; Cymbaluk 1994) and heat stress (Guthrie and Lund 1998). However, horses have a comparatively lower body surface-to-mass ratio (1:90–100 m^2^/kg) than humans (1:35–40 m^2^/kg) which further reduces their ability for heat dissipation (Hodgson et al. 1993). This increases the use of energy required for dissipating accumulated body heat so that body temperature is maintained in the narrow range considered to be normal (Lindinger and Marlin 1995; Brownlow et al. 2016; Hodgson 2014; Tansey and Johnson 2015). To maintain a normal body temperature range, the horse uses four heat transfer mechanisms, thermal radiation, conduction, convection, and evaporation (Fig. 1; Noakes 2008; Guthrie and Lund 1998; Hodgson 2014).

### Thermal radiation

Heat exchange by thermal radiation occurs between the animal’s skin (or hair surface) and the surrounding environment by electromagnetic waves without direct physical contact (Kaviany 2011; Hodgson 2014). Thermal radiation, such as solar radiation or radiation from a fire, is not explicitly physiologically controlled by animals, but it has a significant role in thermoregulation. All physical objects subjected to a temperature above absolute zero (− 273 °C) emit thermal radiation which can be visualized with an infrared camera (Morgan et al. 1997; Meisfjord Jørgensen et al. 2020). A human study has shown that 60% of human body heat can be dissipated by thermal radiation when there is a sufficient thermal gradient under shade (Wendt et al. 2007). The heat gain via radiation becomes greater than the heat dissipation under sunlight even when the ambient temperature is lower than the body temperature, as it depends on the amount of thermal radiation (W m^−2^), added to the ambient temperature (Jessen 2001). Under these circumstances, horses can still maintain their body temperature via other thermoregulatory systems, such as sweat evaporation. However, under hot and humid conditions this way of heat dissipation is limited (Wendt et al. 2007; Cheuvront and Haymes 2001; Holcomb et al. 2014). The colour of the hair coat in horses can also impact body temperature due to differences in solar radiation absorbance (McCutcheon and Geor 2014; Cobb and Cobb 2019). In a study by Cobb and Cobb (2019), it was found that the black and white stripes of the Zebra coat had different temperatures when the animal was standing in full sun. It has been reported that solar radiation absorbance black coat is twice as much as white coat (Maia et al. 2015; Laible et al. 2021), as also reported that the temperature of the black stripes of the Zebra was higher (44 and 56 °C) than the white stripes (36 and 42 °C) (Cobb and Cobb 2019).

### Convection

Convective heat transfer is caused by the movement of a gas or liquid (Guthrie and Lund 1998), such as wind over the skin or breathing air in the lungs. The efficiency of this heat exchange depends on the temperature gradient between the body surface and the surrounding gas or liquid and the viscosity determines how rapidly the warmed gas or liquid is replaced by the cool gas or liquid (Willoughby 2002; McCutcheon and Geor 2014; Jefferson et al. 2018; Kaviany 2014). For example, body heat is transferred from the surface of the horse into the cooler surrounding air and heats up the air (conductive heat transfer), but when the heated air is quickly replaced by wind, the body heat can be dissipated more quickly to the replaced cool air (Mostert et al. 1996; Wendt et al. 2007). As a result, faster gas movement (air and wind) can increase the heat exchange by convection. A horse with long hair has poor body heat dissipation through convection because the hair traps the warmed air and impedes replacement by cooler air (McCutcheon and Geor 2014). The different coat colour of the Zebra can increase convection (Cobb and Cobb 2019). As it was aforementioned in the ‘Thermal radiation’, the black stipe has higher temperature than the white stripe, and the temperature gap, between the warmer air near black stirp and the cooler air near white strip, may cause slight airflow and it increases convective heat transfer (Cobb and Cobb 2019). Also, increased blood flow can help convective heat dissipation via the transfer of the body heat away from working muscle (McCutcheon and Geor 2014) through increased blood flow to the periphery. Increased respiratory rate results in heated air being exhaled from the lungs more quickly and being replaced by cooler air with inhalation. It was reported that a high respiratory rate (> 200 breaths/min) can dissipate 25% of the metabolic heat production of exercising horses (Mejdell et al. 2020).

### Conduction

Conduction is defined as heat transfer through molecular interactions, whereby heat is exchanged between surfaces through contact when the surfaces have different temperatures (Ezekoye 2016; Kaviany 2014). Horses can emit body heat to the surrounding air when the air temperature is lower than body temperature via conduction and convection, but this heat flow can reverse when the air temperature is higher than that of body temperature (Wendt et al. 2007; Kaviany 2014). In regard to convection, the body heat transferred from the surface of the horse to the cooler surrounding air heats up the air, and the heated air can be replaced by wind. This enhances the conductive heat dissipation to the surrounding air (Mostert et al. 1996; Wendt et al. 2007). In horses, skin thickness and hair formation, length and density can affect conductive heat transfer (Guthrie and Lund 1998). The efficiency of conductive heat exchange depends also on relative humidity of the air, as water has a high heat conductivity (Romanovsky 2018). Conductive heat transfer can be the most effective method to cool down the body temperature of horses when water that is cooler than body temperature is applied to the body (Marlin et al. 1998; Takahashi et al. 2020).

### Evaporation

Only equidae, bovidae, and primate species have sweat glands that allow them to use the evaporation of sweat as the primary form of thermoregulation (McCutcheon and Geor 2014). One gramme of water, such as sweat on the skin or water from the respiratory tract, absorbs approximately 2397 kJ of body heat when it is vaporized (Ingram and Mount 1975). Even though both humans and horses use sweat evaporation as a primary thermoregulation method under hot ambient temperature, it was reported that the sweat rate (L/h/m^2^) was three times greater in the exercising horses than in humans in similar exercise intensity (Kingston et al. 1997). Approximately 70% of heat loss from a horse during exercise, is via evaporation when humidity is low (Guthrie and Lund 1998). The sweat of horses, but not humans, is hypertonic and contains abundant Na^+^, K^+^, Cl^−^, and latherin, a protein that decreases surface water tension and makes the sweat spread to help evaporation (Eckersall et al. 1982; Hodgson 2014). Evaporative cooling also occurs via the respiratory tract of horses. Expelled air is always of body temperature with a humidity of 100%. Although horses are not considered to be panting, breathing frequency and the volume of air intake can increase tenfold and up to 18-fold during strenuous exercise relative to that of a horse at rest, and this elevation in breathing enhances evaporative cooling via the respiratory tract (Franklin et al. 2012). In cold circumstances, the respiration rate decreases and becomes deeper in order to decrease heat loss via respiration and maintain gas exchange in the lungs (Mejdell et al. 2020). The efficiency of heat dissipation through evaporation relies significantly on relative humidity, as evaporation is increased when relative humidity is lower due to a difference between the vapour pressure on the body surface and the atmosphere (Geor and McCutcheon 1998; McCutcheon and Geor 2014; Girard et al. 2008). The efficiency of sweat evaporation is further reduced when the conditions are hot and humid, and eventually sweat runs off the horse. The heat loss from the sweat running off the animal is only 5 to 10% of that through evaporation from the skin (Guthrie and Lund 1998; McCutcheon and Geor 2014). Latherin, a protein in horse sweat, makes a bubble-like foam on the skin and this prevents sweat from dripping off the coat thereby enhancing evaporation (Eckersall et al. 1982; Hodgson 2014).

Horses can dissipate accumulated body heat produced by exercise, by increasing conductive, convective, and evaporative heat transfer, increasing heart rate, redistribution of blood flows, respiratory rate and the production of more sweat. However, when both ambient temperature and relative humidity are high, the horse has only a few options to dissipate body heat due to limited, or reversed heat transfer via convection, conduction, or radiation under ambient temperatures higher than their body temperature, and limited evaporative heat transfer resulting from a reduction in heat gradient under high relative humidity (Kaviany 2011; Romanovsky 2018; Geor and McCutcheon 1998; McCutcheon and Geor 2014; Girard et al. 2008). Continuous sweating increases the use of body water and electrolytes which can lead to dehydration (Marlin et al. 1998). Prolonged dehydration can lead to dysfunction of the central nervous system and heatstroke, and, eventually, the horse may die due to a malfunction of thermoregulation (Hodgson 2014).

### Neuroendocrine responses to heat stress

**Fig. 2:**
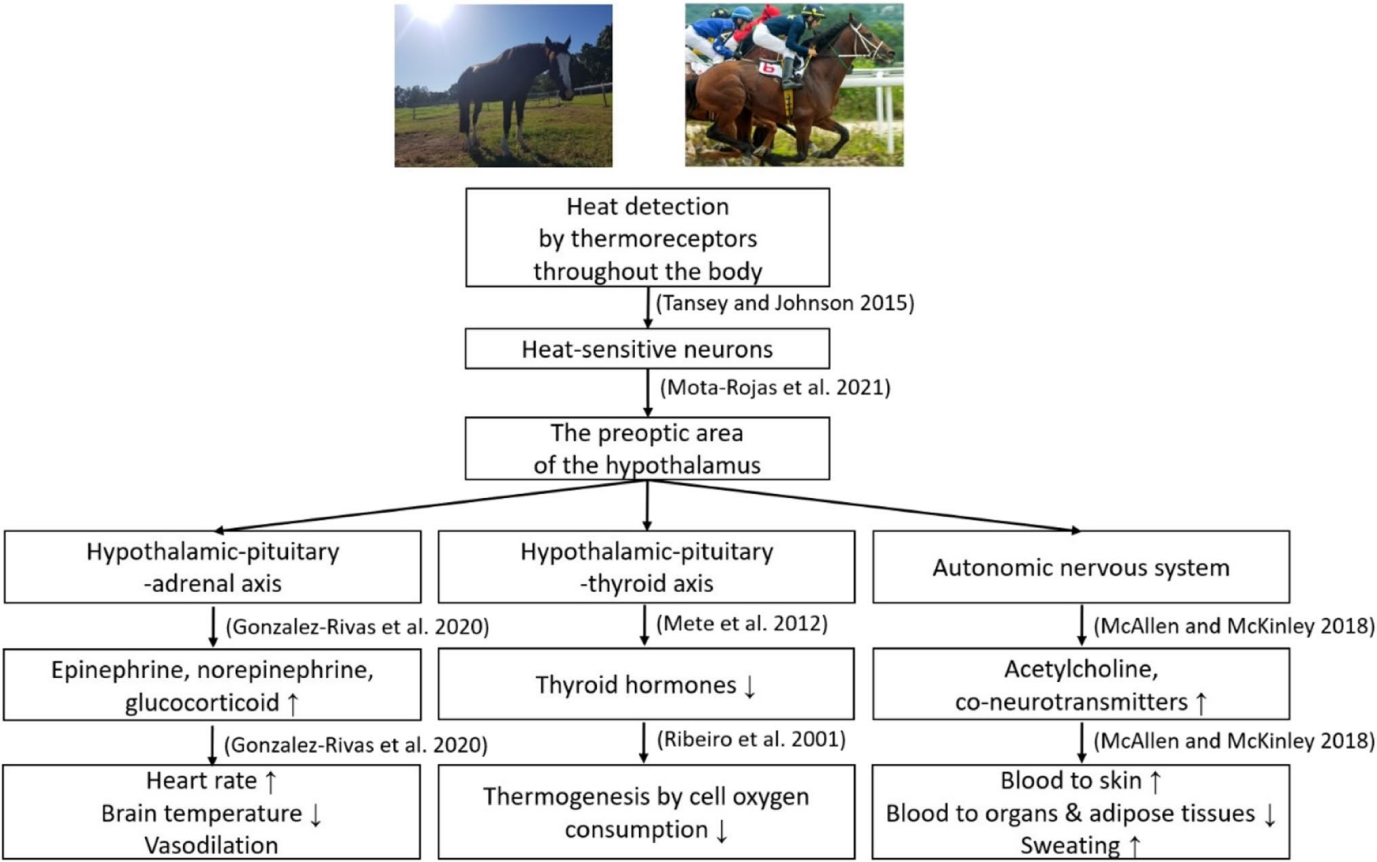
Neuroendocrine and physiological responses to heat stress. Arrows in the boxes — ‘↑’, increasing; ‘↓’, decreasing. The images were adapted from Hyungsuk Kang and open source ‘Adobe stock images’

A change in body temperature to a point outside an animal’s normal range is recognized as a stressor (Pacák and Palkovits 2001) and excessive body heat is detected by the neuroendocrine system which attempts to return body temperature to its normal range (Fig. 2; Gale 1973). The changes in body temperature are detected by the nerve endings (thermoreceptors) of temperature-sensitive neurons in the epidermis, the blood vessels, and the brain, and also from different body systems, e.g. abdominal viscera and spinal cord, of which 20 to 40% detect heat and 5 to 10% detect cold (Lezama-García et al. 2022; Kashio 2021; Gale 1973; Childs 2018). The signals detected by the thermoreceptors are transferred both (1) to the thalamus, and, eventually, to the primary somatosensory cortex in the brain, where they mediate the perception of heat, and (2) to the lateral parabrachial nucleus, and, from there to the preoptic area of the hypothalamus to initiate thermoregulation (Lezama-García et al. 2022; Kashio 2021; Fealey 2013; Childs 2018; Tan and Knight 2018).

When heat stress is detected by the horse, the hypothalamic–pituitary–adrenal (HPA) axis (Gonzalez-Rivas et al. 2020) and the autonomic nervous system (ANS) (Wendt et al. 2007; Gonzalez-Rivas et al. 2020) are upregulated and hypothalamic-pituitary-thyroid (HPT) axis function is suppressed (Mete et al. 2012; Bernabucci et al. 2010) to maintain body temperature and to prevent excessive heat accumulation. As a result of the upregulation of the HPA axis and ANS, hormones and neurotransmitters such as acetylcholine, epinephrine, norepinephrine, and glucocorticoid (cortisol) are secreted, and downregulation of the HPT axis results in a decrease in the concentration level of thyroid hormones to regulate energy metabolism (Ehrlenspiel 2012; Mete et al. 2012). Together these have a significant role in thermoregulation (Febbraio 2001; Christman and Gisolfi 1985; Gisolfi and Christman 1980; Westfall 2009; Dewitt and Grondin 2011).

Epinephrine and norepinephrine increase heart rate (Lin and Pivorun 1986; Wright 2013; Kozyreva et al. 2015), and acetylcholine and other co-neurotransmitters cause cutaneous vasodilation to increase blood flow to the skin which helps to dissipate the heat from the bloodstream to the air (McAllen and McKinley 2018). Meanwhile, blood flow to internal organs and adipose tissue decreases as more blood is supplied to the skin for this heat dissipation (McConaghy et al. 2002).

Glucocorticoid, which is found in hair, blood, faeces, and saliva in animals (Ghassemi et al. 2014; Kovács et al. 2019; Rees et al. 2016; Narayan et al. 2018), will cause vasodilation to help heat dissipation as part of the body’s thermoregulation system (Aggarwal and Upadhyay 2013). Glucocorticoid concentrations follow a diurnal rhythm (Giannetto et al. 2012) but also depend on individual heat tolerance, and are typically lower in animals with high heat tolerance, and higher in animals with low heat tolerance (Follenius et al. 1982). Glucocorticoid concentrations are greater in acute heat stress conditions than in chronic conditions (Gonzalez-Rivas et al. 2020). Sweating, which is a key to heat dissipation in a horse, is initiated by activation of the sympathetic nervous system in the skin. The temperature for the onset of sweating is detected by the thermo-sensitive neurons and the sympathetic nerves signal that initiates sweating (Sugenoya et al. 1990).

The HPT axis secretes thyroid hormones to regulate energy metabolism (Ehrlenspiel 2012). The thyroid hormones result in increased body temperature with increasing oxygen consumption in cells, such as in the mitochondria (Ribeiro et al. 2001). The level of thyroid hormones in serum decreases during 24 h of hyperthermia when the air temperature is high (Mete et al. 2012; Pineda and Dooley 2003). When mammals adapt to warmer climatic conditions over the long term, the level of thyroid hormones is reduced to decrease metabolic thermogenesis (Bernabucci et al. 2010).

### Brain cooling

Horses have guttural pouches and cavernous sinuses that are believed to have the cool-down function of the brain (Baptiste 1998; Baptiste et al. 2000). The two guttural pouches are located in the caudal area of the head and are the largest pouches of any mammals (Baptiste 1998). Blood flows through the internal carotid arteries which traverse the pouches, and is cooled (Maloney et al. 2002; McConaghy et al. 1995). Even under 100% humidity, and when convection heat loss is impaired, the blood flowing to the brain is cooled down due to the cooling effect of the guttural pouches (Baptiste 1998). The cavernous sinuses of the horse have a similar function to the carotid rete in carnivores, but the carotid rete has a relatively smaller surface area and fewer arterial branches compared with the cavernous sinuses in horses (McConaghy et al. 1995; Baptiste et al. 2000). However, another study showed that there was no brain cooling by the guttural pouches during exercise which requires high ventilation rate, nor at rest, and that the only response to the high brain temperature is the initiation of sweating or cessation of exercise (Mitchell et al. 2006).

The sweat on a human head can reduce brain temperature by approximately 0.4 °C via evaporation (McConaghy et al. 1995). It is assumed that the sweat produced by horses on their head has a similar effect (McConaghy et al. 1995). An increase in respiratory dynamics can also decrease the temperature of blood flowing to the brain by water evaporation from the nasal mucosa and upper respiratory tract (Carithers and Seagrave 1976).

## What happens as heat stress develops?

Horses require little or no additional energy to maintain body temperature when not exposed to hot conditions or are exercising (Morgan 1998). However, the balance can be disrupted in some circumstances, such as during high-intensity exercise or prolonged submaximal exercise in hot (air temperature > 30 °C) and humid (relative humidity > 90%) conditions (Hodgson et al. 1994). During strenuous exercise, 80% of the energy consumed by the muscles is released as metabolic heat, which depends on age and health status (Wallsten et al. 2012; Hodgson 2014; Scott 2005), and this leads to an increase in core body temperature of 1 °C/min (Hodgson et al. 1993; Hodgson 2014). The metabolic processes of muscular contraction can be divided into two types, aerobic which is slower but has higher energy efficiency and anaerobic which is faster but has lower energy efficiency and also produces lactate that can cause fatigue (Pösö et al. 2008). Anaerobic metabolism produces up to 7% more heat during the metabolic reaction than aerobic metabolism due to less efficient energy transfer (Scott 2005). Higher intensity exercise requires more rapid energy consumption in the skeletal muscles, so is more dependent on anaerobic metabolism because it is much quicker than the aerobic pathway (Schuback and Essén-Gustavsson 1998; Langlois 1994). It was reported that during endurance events, horses gained almost 100% of energy by aerobic metabolism, while it was only 70% during a 1600–2000 m Thoroughbred race (Pösö et al. 2008).

Hot and humid climatic conditions may exacerbate heat accumulation in the body by restricting heat dissipation (Brownlow and Smith 2021; Brownlow et al. 2016). It has been documented that body temperature can significantly increase as ambient temperature increases (Soroko et al. 2017b, 2017a; Minka and Ayo 2016; Aujard and Vasseur 2001). Humidity will also influence body temperature. As aforementioned, water has high conductivity, so it helps heat dissipation when it is used as a conductive heat transfer but during, and immediately after exercise, the body temperature of horses is significantly higher when humidity is higher, under the same ambient temperature (Kohn et al. 1999).

Under acute heat stress conditions, the respiration rate of some species increases in an attempt to maximize heat dissipation through evaporation, e.g. dogs (Davis et al. 2017; Ledsome et al. 1981), sheep (Srikandakumar et al. 2003), humans (Robertshaw 2006), and cattle (Cardoso et al. 2015; McManus et al. 2014; Gaughan et al. 2008). In studies of cattle and dogs, respiratory rate increased when ambient temperature increased, while the rectal temperature was not significantly increased (Davis et al. 2017; Cardoso et al. 2015; Gaughan et al. 2010; McManus et al. 2014). Although horses are non-panting animals and they can only breathe through the nostrils, an increase in respiratory rate during periods of heat load has been reported (Kohn and Hinchcliff 1995) that indicate it has a primary role in thermoregulation that can contribute to brain cooling (Robertshaw 2006; Lekeux et al. 2014).

Acute heat stress can affect reproductive functions in both the stallion and the mare. When the body temperature of a stallion is elevated, the scrotal temperature can also be elevated, which may result in poor spermatogenesis or mutations in gamete DNA, as well as decreasing testosterone levels for a few weeks after heat shock exposure (Hansen 2009; Love and Kenney 1999; Setchell 2006). It has also been reported that semen concentration, number of spermatozoa and motile sperm per ejaculation in bulls were lower during summer than in winter and spring (Bernabucci et al. 2010). However, mild heat stress may not result in diminished breeding ability due to the thermoregulation function of the scrotum where there is heat exchange between highly coiled arteries and veins around the testis (Gordon et al. 2014; Amann 2011). In a study of mammalian females, acute heat stress decreased maternal blood flow to the placenta (Alexander et al. 1987) and reduced follicular volume (Wolfenson et al. 1997), which can cause poorer reproductive results.

If environmental heat stress is prolonged by seasonal or geographical location, various physical factors can change, such as normal body temperature range, fat deposition, coat thickness, or hair density, as an adaptation to mitigate the effects of long-term heat stress conditions (Bernabucci et al. 2010; Collier et al. 2019). Furthermore, the sensitivity and population of the receptors for homeostatic signals can be changed, such as by decreasing catecholamines and glucocorticoids (Bernabucci et al. 2010; Collier et al. 2019). When heat stress is prolonged, following changes have been reported such as damage to oocyte quality (Al-Katanani et al. 2002), suppression of gonadotropin-releasing hormone (Satué et al. 2021), reduced numbers of gonadotropin receptors (Hansen 2009; Shimizu et al. 2005) and medium-sized follicles (Roth et al. 2000), impaired embryonic development (Ealy et al. 1993; Bernabucci et al. 2010), and abnormal foetal development (Mortensen et al. 2009; Smith et al. 2012; Yu et al. 2022). In various studies, the heat stress is also recognized as a teratogen (Graham 2005; Ouellet et al. 2021; Barrier et al. 2009). Adaptation to heat stress changes the sensitivity of the onset of sweating, as well as the number of active sweat glands and its volume (Sawka et al. 2001; McCutcheon and Geor 2000; Sneddon et al. 2008). It was reported that repeated exercise initiates the onset of sweating at lower body temperature (McCutcheon and Geor 2000) and the sweat gland volume in Thoroughbreds was significantly increased during the summer season when compared to the winter season (Sneddon et al. 2008). Adaptation to the prolonged heat stress can be fixed in gene expressions such as changes in morphological traits, behaviour, metabolism, and productivity over generations to decrease metabolic heat production and increase heat dissipation efficiency (Geor et al. 1996; Sejian et al. 2018; Bernabucci et al. 2010).

### Heat stress–related illness

Heat stroke is a life-threatening illness that is caused by central nervous system dysfunction (Leon and Helwig 2010) and a horse with heat stroke will show depression, weakness, refusal to work, decreased appetite, tachypnoea, tachycardia, elevated rectal temperature (41 to 43 °C), lethargy, poor sweating response (hot and dry skin), and slow capillary refill response (muddy mucous membranes) (Padalino et al. 2017; Orsini and Divers 2014). In humans, heat stroke is seen when climatic conditions are hot and humid or while performing strenuous work or exercise (Peiris et al. 2017; Leon and Helwig 2010). It can change mental status (headache, confusion, or coma), as well as damaging the brain, liver, kidneys, and muscles (Peiris et al. 2017). Similar effects and even coma and death can be caused in humans by heatstroke (Leon and Helwig 2010). Heatstroke in horses may occur when the rectal temperature exceeds 41 °C as a result of over-exercise during hot and humid conditions; being confined in a space where the ventilation system is inadequate; or being moved to a hot and humid climate from a cool climate without an acclimatization period (Hines 2018; Orsini and Divers 2014). It has been reported that the temperature inside a horse float maybe 5.1 to 9.5 °C higher than the outside temperature (Purswell et al. 2010), and, in addition, the fear level of a horse in a float can cause heat stress and over-sweating, even in comfortable climate conditions (Padalino et al. 2016). It has been reported that 10% of horse transport–related incidents are heatstroke-related (Padalino et al. 2016).

Anhidrosis is a reduction or lack of sweat and is normally seen in exercising athletic horses or stabled horses exposed to a hot and humid climate for long periods (Johnson et al. 2010; Divers 2014). The exact cause of anhidrosis is currently unknown, but it is thought that prolonged exposure to hot and humid conditions may decrease the sensitivity of the sweat glands to epinephrine resulting in a decrease or total cessation of sweating (Jenkinson et al. 2007; Johnson et al. 2010). Horses with anhidrosis can present with exercise intolerance, hyperthermia, reduced appetite and water intake, higher rectal temperatures and respiratory rate, and depression (Johnson et al. 2010; Divers 2014). The clinical signs generally develop gradually; however, in some cases, there is rapid onset. The symptoms can be ameliorated by the administration of an antipyretic agent, providing electrolyte supplementation, clipping body hair, or moving the horse into an air-conditioned stall (Orsini and Divers 2014).

As sporting horses do high-intensity exercise during a 3-day event (average of 4.5 m/s of speed with an increase of 0.30 °C of body temperature per minute), endurance racing (average of 5.0 m/s of speed with an increase of 0.22 °C of body temperature per minute) or Thoroughbred racing (average 16.0 m/s of speed with increase 1.00 °C of body temperature per minute) (Hodgson et al. 1994), their body temperature can exceed a critical range rapidly in hot and humid climates, and they can suffer from exercise-induced heat stress, which is called exertional heat illness (EHI) (Takahashi and Takahashi 2020; Brownlow and Smith 2021). In most cases, EHI occurs suddenly because of the rapid production of metabolic heat during high-intensity exercise and it can lead to hyperthermia, CNS dysfunction, or aggressive behaviours (Brownlow et al. 2016). Rapid detection of early-stage EHI after exercise followed by aggressive interventions is essential to prevent the progression of the disorder (Brownlow et al. 2016).

## How is heat stress identified?

Signs of heat stress in horses include rapid shallow breathing, flared nostrils, unpredictable behaviour and gait, very high body temperature, high respiratory rate, high heart rate and profuse sweating (Pritchard et al. 2006; Equestrian Australia 2017; Brownlow et al. 2016). Measuring body temperature can provide a quick and easy method to detect heat stress in horses. Several studies have attempted to define heat stress or hyperthermia in horses; however, there are variations in regard to how the critical core temperature was assessed (Hodgson et al. 1993, 1994; Guthrie and Lund 1998; Jones et al. 1989). Measuring the body temperature of horses using a rectal thermometer is the most commonly used method because it is quick and easy to use. However, it is only a point-in-time measurement and does not reflect continuing changes in body temperature.

Continuous body temperature measurement and its relationship with clinical signs of heat stress may improve horse welfare and management of heat-related diseases or heat-affected horses (Green et al. 2005, 2008; Ramey et al. 2011). Several methods can be used for continuous measurement of body temperature, such as the placement of a thermocouple in the rectum (Verdegaal et al. 2017); a central-venous catheter for obtaining blood temperature (Lund et al. 1996); a gastrointestinal thermal sensing pill (Verdegaal et al. 2014); thermal sensing microchips (Kang et al. 2022) when placed in muscle sites that are reflective of internal body temperature; and infrared thermography (Valera et al. 2012) which can measure eye temperature and thermography camera (Soroko et al. 2018) to obtain body surface temperature.

### Rectal temperature

Rectal temperature is one of the easiest measurements for determining body temperature in many animal species (Ritter et al. 2009; Burfeind et al. 2010; Ramey et al. 2011; Dangarembizi et al. 2017; Robinson et al. 1998; Romano et al. 1993) and the use of a rectal thermometer is the most commonly used method of obtaining body temperature in horses (Hall et al. 2019; Hine et al. 2015; Giannetto et al. 2012). The rectal temperature has been shown to be highly correlated with deep core body temperature in some horse studies (Morgan 1997; Marlin et al. 1999; Collins et al. 2016; Ramey et al. 2011). However, other studies have shown that it is not ideal for the detection of the early stages of fever (Maeda and Oikawa 2019) or for monitoring body temperature during exercise or immediate post-exercise (Kang et al. 2020). Also, some horses do not tolerate the procedure well when they are suffering from heat stress, thereby presenting an occupational safety risk, and there is also a risk of disease transfer and other hygiene issues when using this method (Johnson et al. 2011; Dangarembizi et al. 2017). Moreover, the depth of the thermometer in the rectum influences the rectal temperature measurement and does not allow continuous assessment (Hall et al. 2019).

### Eye temperature

Eye temperature measurement using infrared thermography has some advantages as it is a passive, non-invasive, and rapid method (Johnson et al. 2011). It has also been used in several studies of horses, cattle, and pigs, and positive correlations were found between eye temperature and heat stress (Johnson et al. 2011; Church et al. 2014; Soroko et al. 2016; Petry et al. 2017; Stewart et al. 2007; Valera et al. 2012). Eye temperature can also be used to detect fever and stress-induced hyperthermia in humans (Johnson et al. 2011). However, even though eye temperature can detect fever, there is little data to support its relationship with the core body temperature of horses during exercise or recovery from hyperthermia.

### Gastrointestinal pill

The ingestible telemetric temperature pill can be used in horses to record body temperature continuously and non-invasively (Verdegaal et al. 2017). However, in horses it must be administered by nasogastric intubation (Verdegaal et al. 2021, 2017). Once the pill is administered, the pill remains in the system for approximately 12 days before being expelled through faeces, but the transit time differs depending on age, diet, gender, or physical status and the body temperature detectable days may be terminated within 5 days (Verdegaal et al. 2017). It has been reported that the pill temperature correlated well with rectal temperature (O'Brien et al. 1998; Wilkinson et al. 2008; Sparling et al. 1993). However, it also needs more experimental time than others because it does not begin to correlate with the rectal temperature until at least 3 h after being administered (Roache 2010). To activate the temperature recording using the pill, it is recommended that the pill be ingested up to 6 h prior to use (Bongers et al. 2015). Furthermore, this pill is expensive and can only be used once, unlike other techniques such as monitoring rectal temperature or tympanic temperature (Bongers et al. 2015).

### Blood temperature

Central venous temperature is assumed to be the most accurate site for measuring core body temperature due to its proximity to the heart and because it receives blood from the entire body (Hayes et al. 1996; Furlong et al. 2015). As central venous blood temperature has a significant correlation (r = 0.652, *p* < 0.001) with brain temperature (Nicholson and Iserson 1991), it is an ideal means for monitoring the temperature of the brain which is more vulnerable to heat stress than the other organs and can cause the death of an animal by biochemical changes resulting from hyperthermia (Burger and Fuhrman 1964; McConaghy et al. 1995). It is widely used as an indicator of core body temperature in humans (Furlong et al. 2015; Lefrant et al. 2003; Abo-Salem and Ramadan 2015; Giuliano et al. 1999). However, it is not practical in horses because it requires an invasive procedure for insertion of a catheter with a thermistor into the jugular vein (Whitener et al. 2014), and is difficult to keep in place.

### Muscle temperature

Muscle temperature has been used in several exercise and heat stress studies but requires invasive procedures if using the technique which requires the insertion of a needle for placement of a thermocouple wire into the muscle (Weishaupt et al. 1996; Hodgson et al. 1993). Recently, new technology using percutaneous thermal sensing microchips has been investigated (Rey et al. 2016; Iyasere et al. 2017; Robinson et al. 2008; Reid 2014; Auclair-Ronzaud et al. 2020; Torrao et al. 2011). The use of thermal microchips allows non-invasive measurement of muscle temperature once the microchip is implanted. Relationships between core body temperature and the microchip temperature obtained from muscles have been assessed in several studies (Weishaupt et al. 1996; Auclair-Ronzaud et al. 2020; Robinson et al. 2008; Kang et al. 2020, 2022). It was found that muscle temperatures measured using the microchip during exercise and the cooling down phase had significant positive correlations with core body temperature (Kang et al. 2020). However, it varies according to the muscle in which it is inserted (Kang et al. 2020; Torrao et al. 2011) and so it is important that a reliable muscle location for insertion of the microchips be determined for horses.

## Methods for mitigating severe heat stress

It has been reported that the jugular vein blood temperature, abdominal temperature, and rectal temperature of horses undertaking treadmill exercise (from 3 m/s up to 4.5 m/s for 30 min) in a thermal comfortable room (max room temperature 16 °C and humidity 75%) increased from 36.2 °C, 37.4 °C, and 37.6 °C at rest, to 38.4 °C, 40.6 °C, and 39.9 °C, respectively (Weishaupt et al. 1996). However, without appropriate interventions, the abdominal and rectal temperature continued to increase to 41.5 °C and 40.1 °C, respectively, during the first 10 min of recovery time in the same space, and the abdominal temperature was still 0.7 °C (38.1 °C) higher than the resting level (37.4 °C) after an hour under the same room conditions (Weishaupt et al. 1996). Effective and practical intervention methods include using a fan to supply airflow or pouring cool water on the skin can be applied to minimize the prolonged heat stress (Jeffcott et al. 2009; Williamson et al. 1995; Takahashi et al. 2020; Brownlow 2018; Marlin et al. 1998).

In a study of human body temperature, it was found that using a fan for cooling was more effective in reducing core body temperature than ice-cooling because the air movement from the fan enhances evaporation heat loss from the skin (Hamada et al. 2006). The participants in the study answered that they felt more comfortable when the body cooling was with fanning than without fanning because there was less physiological activity required to supply more blood to the skin (Jay et al. 2019). Furthermore, cooling with fans can help convection heat transfer between skin and air. Even though the rectal temperature did not show a significant difference, the hot fan cooling made the people more comfortable, which meant they had greater tolerance against the heat when fanning exists (Jay et al. 2019). Fanning (ventilation) also helps cool down horse core body temperature (Takahashi et al. 2020). However, this method may not be as efficient as cold water cooling under hot and humid climate conditions, because of limited evaporative heat dissipation by high humidity and higher conductive heat transfer from the body to cold water by a greater temperature gradient between the two.

Cold water cooling is a strategy that is probably the easiest and most popular method to cool down a hyperthermic horse. It can increase conductive heat dissipation where the temperature gradient between the skin and the water is greater. This method is also used as a gold standard for heatstroke treatment in humans (Casa et al. 2007). Marlin et al. (1998) reported that when horses were cooled with cold water (6 °C) coupled with scraping the water off 6 times with 30-s intervals, the core body temperature (blood temperature measured by a thermistor via a left jugular vein) dropped 4.1 °C in 11 min, while rectal temperature and muscle temperature dropped 1.1 °C and 2.0 °C, respectively. A similar response has been seen in antelope and human hyperthermia studies. When the antelopes were doused with cold water (4 to 17 °C), the body temperature dropped three times faster than the no intervention group (Sawicka et al. 2015) and the human study showed an immediate drop in rectal and skin temperature from 39.55 °C to 37.55 °C and from 36.91 °C to 13.05 °C (Gagnon et al. 2010).

However, the strategy used is important as it is known that cooling horses using cold water (poured on), scraping after pouring cold water, or continuously pouring tap water give different outcomes (Takahashi et al. 2020). In a recent study, it was revealed that applying cold water itself has a greater cooling down effect on its own, than when it is followed by scraping the poured water (Takahashi et al. 2020).

Air movement and water application can help overheated horses to thermoregulate appropriately. By combining the two cool-down strategies, the cool-down effect may be maximized. Methods such as a misting fan, hosing cold water, and then exposing horses to a fan will enhance the cooling rate (Brownlow 2018), as it increases conductive heat dissipation using water that has high conductivity and convective heat dissipation. The cooling rate will increase further with a larger quantity of water and a stronger velocity of the fan (Gaudio and Grissom 2016), but only up to a point.

Providing shade is an important component for mitigating heat stress in horses during hot climatic conditions by minimizing heat accumulation caused by solar radiation. Horses spent more time in shady areas during peak solar radiation (Holcomb et al. 2014; Holcomb and Stull 2016). When the horses were exposed to direct solar radiation without shade, they were found to spend more time near the drinking water and splashing the water to wet their skin, therefore may be as an adapted mechanism to take advantage of water evaporation on the skin (Holcomb et al. 2013). The preferred behaviour is dependent on the individual horse (Janczarek et al. 2021). Physiological traits showed that the horses without shade had a higher respiratory rate, rectal temperature, sweating rate, and skin temperature (Holcomb et al. 2013).

## How do we prevent heat stress?

Although interventions to cool down heat-stressed horses are effective, it would be better if the horses were not heat stressed. Accurate measures of climate conditions may help to improve the management of the horse and protect them from severe heat stress.

Several thermal indices have been used to measure thermal environments in livestock industries and international sports games, such as the wet-bulb globe temperature (WBGT), the temperature-humidity index (THI), a wet-/dry-bulb temperature index (WD index), the heat load index (HLI), and effective temperature (ET) (Gaughan et al. 2008; DeShazer 2009; Bjerg et al. 2018; Lallo et al. 2018). Among the indices, WBGT is used as a thermal index for many international human and horse events, such as equestrian sports run by Fédération Equestre Internationale (FEI), and many international sports games (Olympic Games, Fédération Internationale de Football Association (FIFA), International Amateur Athletic Federation (IAAF), and International Tennis Federation (ITF)) (Schroter et al. 1996; Brocherie and Millet 2015). The WBGT index was originally developed for human training operations in the army, but it was officially adopted by FEI to use as a thermal index in the equestrian events in the 1996 Olympic games (Schroter et al. 1996).

The WBGT can be calculated for indoor (Eq. 1) and outdoor (Eq. 2) activities:

1 $$WBGT=\left(0.7*Tnw\right)+(0.3*Tg)$$

2 $$WBGT=\left(0.7*Tnw\right)+\left(0.2*Tg\right)+(0.1*Ta)$$

where Tnw is natural wet-bulb temperature, Tg is the black globe temperature, and Ta is air temperature (Patel et al. 2013). The WBGT thresholds and activity recommendations for sports activities in humans and equines are presented in Table 1 (Schroter et al. 1996; Blazejczyk et al. 2012). The WBGT is recommended as a heat stress index due to its convenience and comprehensiveness, but the limitation is the lack of adjustments for humidity and air movement (Budd 2008) which should also to be included.

**Table 1:** Wet-bulb globe temperature (WBGT) and recommendations of sports activity in human and horse

WBGT	Activity recommendations	
	Human (Blazejczyk et al. 2012)	Horse (Marlin et al. 2018; Schroter et al. 1996)
< 18	Unlimited	No changes to the FEI recommended format should be necessary
18–23	Keep alert for possible increases in the index and for symptoms of heat stress	
23–28	Active exercise for unacclimatized persons should be curtailed	
28–30	Active exercise for all but the well acclimatized should be curtailed	Some precautions to reduce the heat load on horses are advised
30–32	All training should be stopped	Additional precautions to those above to limit overheating of horses will be necessary
32–33		In these climatic conditions, further modification of the course will be necessary
> 33		These climatic conditions may not be compatible with safe competition

Additional precautions should be put in place such as the preparation of additional ice, water, sponges, towels, chiller bins, and bags to assist in the cooling of horses. Also, additional veterinarians to monitor horses and adequate ventilation with fans are also recommended (Racing New South Wales 2018; Racing Victoria 2014; Racing Queensland 2016). When the climatic conditions are extreme (ambient temperature over 38 °C with humidity exceeding 20% or over 29 °C by WBGT), the race meeting can be modified, postponed, or cancelled (Racing New South Wales 2018; Racing Victoria 2014; Racing Queensland 2016).

## Implications for the future

Heat stress is a serious welfare issue for horses as it can cause severe illness and death. Horses can dissipate accumulated body heat by physical reactions, such as conduction, convection, radiation, or evaporation, and physiological responses, such as secreting stress hormones or producing sweat. However, when the heat load is prolonged or exceeds the heat stress tolerance due to high-intensity exercise during hot and humid conditions, abnormal organ function, impaired immune system, or heat stroke may occur (Marklund et al. 2004).

As discussed, heat-stressed horses can be cooled down by pouring cool water over the horse either alone or combined with air ventilation where possible. However, there is currently no accurate information regarding the most effective cool-down method, e.g. the quantity of water required, the temperature of water applied, and the delivery system of water over the horse’s body. This information will help to improve the treatment of horses when they are heat stressed.

Obtaining an accurate body temperature measurement of horses can help to detect heat stress in the early stage, allowing timely interventions which will prevent adverse heat-related outcomes. There have been several efforts to measure accurate body temperature quickly, safely, and non-invasively. However, there is a lack of any standardized method or validated interpretation of heat stress in horses. Furthermore, developing and providing proper guidelines and educating horse owners and trainers can improve the welfare of horses by protecting them from heat stress. Therefore, further research is required to define the relationship among the body temperatures of horses, climate conditions, and the physiological responses in diverse circumstances. This information will provide more accurate guidance for the treatment and prevention of heat stress under different circumstances.

## Data Availability

Data sharing not applicable to this article as no datasets were generated or analysed during the current study.
